# Quantification of Free-Living Community Mobility in Healthy Older Adults Using Wearable Sensors

**DOI:** 10.3389/fpubh.2018.00216

**Published:** 2018-08-13

**Authors:** Patrick Boissy, Margaux Blamoutier, Simon Brière, Christian Duval

**Affiliations:** ^1^Orthopedic Service, Department of Surgery, Faculty of Medicine and Health Sciences, Université de Sherbrooke, Sherbrooke, QC, Canada; ^2^Research Centre on Aging, CIUSSS Estrie CHUS, Sherbrooke, QC, Canada; ^3^Department des Sciences de l'activité Physique, Université du Québec à Montréal, Montreal, QC, Canada; ^4^Research Center, Institut Universitaire de Gériatrie de Montréal, Montreal, QC, Canada

**Keywords:** GPS, accelerometers, signal processing, wearability, usability, activity space measures, lifespace measures

## Abstract

**Introduction:** Understanding determinants of community mobility disability is critical for developing interventions aimed at preventing or delaying disability in older adults. In an effort to understand these determinants, capturing and measuring community mobility has become a key factor. The objectives of this paper are to present and illustrate the signal processing workflow and outcomes that can be extracted from an activity and community mobility measurement approach based on GPS and accelerometer sensor data and 2) to explore the construct validity of the proposed measurement approach using data collected from healthy older adults in free-living conditions.

**Methods:** Personal, functional impairment and environmental variables were obtained by self-report questionnaires in 75 healthy community-living older adults (mean age = 66 ± 7 years old) living on the island of Montreal, QC, Canada. Participants wore, for 14 days during waking hours on the hip, a data logger incorporating a GPS receiver with a 3-axis accelerometer. Time at home ratio (THR), Trips out (TO), Destinations (D), Maximal distance of destinations (MDD), Active time ratio (ATR), Steps (S), Distance in a vehicle (DV), Time in a vehicle (TV), Distance on foot (DF), Time on foot (TF), Ellipse area (EA), and Ellipse maximum distance (EMD) were extracted from the recordings.

**Results:** After applying quality control criteria, the original data set was reduced from 75 to 54 participants (28% attrition). Results from the remaining sample show that under free-living conditions in healthy older adults, location, activity and community mobility outcomes vary across individuals and certain personal variables (age, income, living situation, professional status, vehicle access) have potential mitigating effects on these outcomes. There was a significant (yet small) relationship (rho < 0.40) between self-reported life space and MDD, DV, EA, and EMD.

**Conclusion:** Wearability and usability of the devices used to capture free-living community mobility impact participant compliance and the quality of the data. The construct validity of the proposed approach appears promising but requires further studies directed at populations with mobility impairments.

## Introduction

Mobility is a fundamental part of both basic activities of daily living and instrumental activities of daily living occurring in the home. It can be defined in space and time as the ability to move oneself (e.g., by walking, using assistive devices, or transportation) within community environments that extend from one's home to the neighborhood and regions beyond (1). Community mobility is indispensable for accessing services and products as well as participating in social, cultural, and physical activities (2). Cross-sectional and longitudinal studies have shown the positive and negative impacts of community mobility on quality of life and mortality (3–6). The importance of community mobility for older adults is now more than ever recognized (4), as data from the most recent National Health Interview Survey in the USA report that almost 20% of those over the age of 65 years have reported difficulty with mobility-related activities such as walking a quarter mile (7). Understanding determinants of community mobility disability is critical to developing interventions aimed at preventing or delaying disability in older adults (8) To better our understanding of these determinants, capturing and measuring community mobility is a key factor.

Mobility at large has traditionally been studied using self-report questionnaires on the perceived ability and capacity of individuals (i.e., what an individual could do or has done) or through performance-based tests in clinical settings (9). While some of these instruments provide an acceptable illustration of the relationship between perceived ability, capacity, and performance measures of function (10), these measures do not necessarily translate into actual real-life performance (11). Several studies have shown that they in fact remain a poor proxy of real life mobility of an individual, as they fail to capture the dynamics between the environment, the intrapersonal factors of mobility restriction and the real life expression of this mobility (12). This is due to their nature of being indirect assessments (i.e., an evaluation of functional capability under controlled experimental conditions) of a realistic enacted function that is modulated by complex interactions between internal physiologic capacity, motivation, and external challenges older adults experience in daily life.

Activity space, a concept originating from medical geography and defined as “the local areas within which people move or travel in the course of their daily activities” (13) has been used to examine how people's habitual movements affect their interactions with their environment in healthcare accessibility studies (14), exposure studies (15, 16), and evaluations of the built environment (17). In recent years, the measure of an individual's lifespace (an adaptation of the activity space concept) has been proposed as a better way to capture both the functional and psychological aspects of mobility while offering a better reflection of actual mobility performance. Lifespace can be defined as the size of the spatial area in which a person purposely moves through in daily life, as well as the frequency of travel within a specific time frame (18). Lifespace is also a measure of where a person goes, the frequency of going to these locations, and their dependency in getting there (19). Thus, this measure not only captures the actual spatial extent of movement, but also the desire for movement and being involved in the larger social environment. As such, a constricted life space may be a consequence of poor health, as a result of impaired sensory, motor, or cognitive functioning, that consequently makes it difficult to move throughout the community at the same level as an individual who is healthy (20). Greater lifespace has additionally been found to be correlated with better global cognition (21). Lifespace has been found to be negatively correlated with age and positively correlated with years of education (20) while men tend to have a larger life space than women (18, 20, 22).

Lifespace can be measured using various different instruments (23–29). The majority of these instruments rely on a self-report questionnaire. Amongst those, the Life-Space Assessment (LSA) proposed by Baker et al. (19) is the most widely used questionnaire instrument for assessing lifespace in community dwelling elders. The LSA permits assessment of the full range of mobility and documents how far and often a person travels to each of the defined levels, while also considering the use (or lack of) assistance. It measures a person's usual pattern of mobility during the month preceding the assessment.

With the introduction and use in research of miniaturized body worn sensing systems incorporating motion sensors, physiological sensors, and location sensors it is now possible to collect and store data on different aspects of human movement and behaviors under free living conditions for long periods of time (30, 31). Of particular interest is the fusion of such sensor technology. Geotracking (Global Positioning System-GPS) and motion sensing (inertial sensors) technologies have been used in older adults to characterize and measure physical activity location, travel behavior and modes of transportation, neighborhood walkability, and overall lifespace (32–50). With regards to these technologies and the proposed outcomes studied in the literature, details describing how these outcomes are extracted and compiled as well as their construct validity are often missing. Part of the challenge in the use and fusion of these technologies are creating meaningful aggregations of space, time, and behaviors from raw sensor data cumulated over multiple days. The datasets generated per individual is substantial, the data sources are not clean and require multiple computational steps. As stated by Jankowska et al. (51) in her proposed framework for the use of GPS, GIS, and accelerometry, the development of better methodologies that can fully make use of these rich data is needed. The objectives of this paper are to (1) present and illustrate the signal processing workflow and outcomes that can be extracted from an activity and community mobility measurement approach based on GPS and inertial sensing; (2) explore the construct validity of the proposed measurement approach from data collected in healthy older adults in free-living conditions.

## Materials and methods

### Participants and protocol

This study used an exploratory cross-sectional design. A non-probabilistic sample of 75 healthy older adults, aged 55–85 years of age, was recruited by convenience sampling using posted advertisements and an existing bank of participants of the Research Centre of the Institut Universitaire de Geriatrie de Montréal, Quebec, Canada. The inclusion criteria consisted of being in healthy condition and residing on the island of Montreal. The exclusion criteria were a Body Mass Index (BMI) over 30, having co-morbidities affecting mobility, using a walking assistive device or having discomfort or difficulty walking during any daily living activity. The study was approved by the Ethics Review Board of the Research Center of the Montreal Geriatric Institute and all participants provided informed written consent to participate.

**Figure 1:**
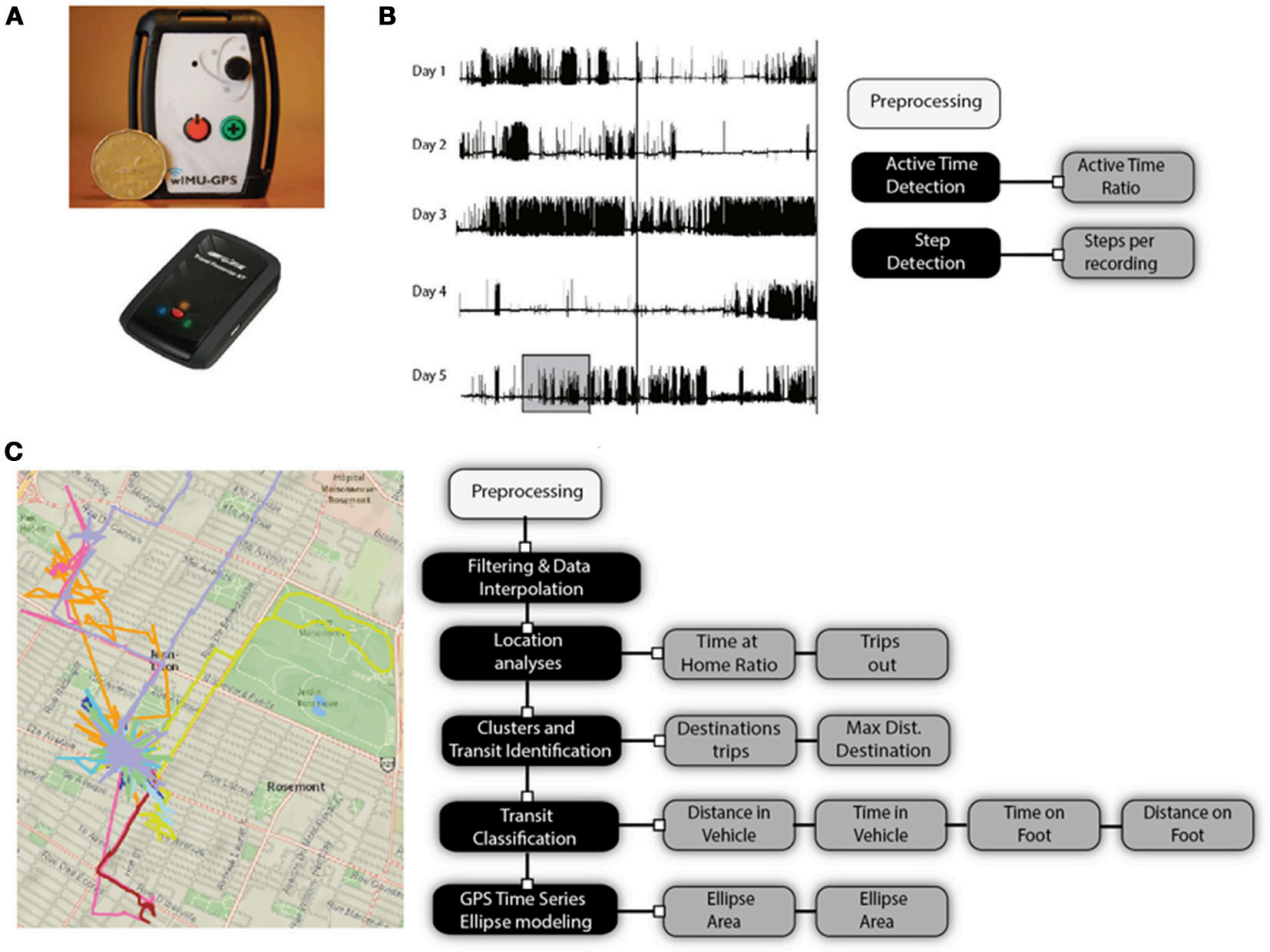
**(A)** Wireless inertial unit with GPS (WIMU-GPS) and quartz GPS data logger; raw signals and signal processing and analysis of **(B)** acceleration, and **(C)** GPS signals.

For 14 days, each participant wore a wearable data logging system at the waist (using a belt or clip on their belt) during waking hours (from the time they dressed to bedtime) (Figure 1A) which incorporated either a GPS receiver unit (Q-Starz travel XT recorder (52) or a GPS receiver unit with an inertial sensor (WIMuGPS-Wireless Inertial Measurement unit with GPS (53). Participants were required to charge the device each night and were instructed not to change their lifestyle or habits. During the study, three meetings with the experimental team were scheduled on days 1, 7, and 14. The first two meetings took place at the participant's home. The objective of the first meeting was to initiate the protocol (consent, instruction on the use of the wearable data logging system) and to obtain anthropometrical measures. The objective of the second meeting was to download data from the data logger system and ensure participant's compliance and comfort. In the final third meeting, a performance evaluation and self-report questionnaire were completed at the Department of physical activity sciences, Université du Québec à Montréal (UQAM), Montreal, QC. Canada. Activity and mobility outcomes from data recorded during days 1, 7, and 14 which excluded these visits and activities related to the study, as they were removed from the dataset as a means to eliminate any effect on the mobility and activity profiles of the participants. The data collection took place from May 2012 to October 2013 to prevent bias from seasonal effects related to winter (54).

### Variables and outcome measures

#### Personal, functional impairments, and environmental variables

Personal variables were assessed by a self-report questionnaire administered on day 1 and included: *age* (coded to categorical variable: < or > 75 years old), *gender* (coded to categorical variables: male or female), *education level* in years of schooling (coded to categorical variables: < or > high school level), *total annual income* of the household in $ (coded to categorical variables as low income or > low income based on the Statistics Canada low-income status cut offs for Census Metropolitan Area > 500 000 inhabitants or more) (55), *living status* (coded to categorical variables: alone or with someone), *professional status* (coded to categorical variables: retired or working), *vehicle access* (coded to categorical variables: access to a car, no access to a car).

Functional impairment variables included *body composition, pain, cognition and depression* which were also assessed on day 1. Body composition was assessed using the *Body Mass Index* (BMI), calculated by dividing the weight by height in meters squared (kg/m^2^). Height in centimeters (cm) was measured in standing position without shoes using a measuring rode (Seca® model 213) and the participants were weighed without shoes in kilograms (kg) on a weighing scale (Adam® GFK660a). BMI values were coded into categorical variables: overweight or normal based on the National Heart, Lung, and Blood Institute 25 kg/m^2^ threshold value (56). Pain in the last 30 days was assessed by questioning their degree of activity limitations due to pain on a four-point Likert scale. The question was taken from the Health Utilities Index (57) and Likert scores were coded to categorical variables: having pain or not having pain in the last 30 days. Cognitive impairments were assessed using the Montreal Cognitive Assessment (MoCA). The MoCA (58) uses a series of tasks and questions to assess the following cognitive domains: attention and concentration, executive functions, memory, language, visuoconstructional skills, conceptual thinking, calculations, and orientation. The sub scores for each section were summed for a possible maximum of 30 points. The total score was coded into categorical variables: cognitive impairments or no cognitive impairments, using the established 26-point cut-off proposed (58). Lastly, depression was assessed using the Geriatric Depression Scale (59). The total score was coded to categorical variables as having depressive symptoms or not having depressive symptoms based on the established 10-point cut-off (59).

Thirdly, the environmental variables consisted of the *perceived proximity to neighborhood services and amenities* (resources) and was assessed using a series of 12 questions from Levasseur et al. (60). For each question, participants were asked to estimate the waking distance (in minutes) from their home to each specified nearest resource. The 12 resources cited included: a grocery/food store, convenience/corner store, bank, pharmacy, community/leisure center, sports center, restaurants/bistro/café, library/cultural center, store/shopping centers, church/place of worship, CLSC/medical clinics, and parks. The average walking time across these 12 resources was used as the outcome for this environmental variable. The average walking time in minutes was coded to categorical variables: < or > than 15 min using the 2009 National Household Travel Survey (NHTS) data as a reference. Finally, the lifespace of the participants was evaluated using the lifespace questionnaire (27). The total score out of 120 was computed as the outcome.

#### Free living activity and mobility measures

Activity and mobility variables were measured non-simultaneously with two wearable systems with data logging capabilities: the Q-Starz travel XT recorder (52) and the WIMuGPS-Wireless Inertial Measurement unit with GPS) (53). An overview of these two wearable systems used, raw signals, signals processing steps and extracted outcomes from continuous daily recordings for activity and mobility are presented in Figure 1.

Raw accelerometer signals are processed to determine active time periods and compute the number of steps taken during active time periods. Recordings from the GPS receiver are processed to create individual daily GPS coordinate (longitude, latitude, altitude) time series. The time series are filtered, interpolated and processed to extract location, establish if they occur in clusters or in transit between clusters and classify the type of transit: in a vehicle or on foot. The specifics of the signal processing steps applied to accelerometers and GPS data collected are described in more details in Tables 1, 2 respectively. Briefly, raw signals from accelerometers during multiple days are preprocessed and processed (black boxes) using active time detection according to thresholds and a specific temporal window while steps are counted using a two-phase step detection algorithm. GPS data are filtered based on device-reported data precision and physically impossible instantaneous velocity between continuous data points. Interpolation on missing GPS data is performed to build a complete timeseries without any temporal hole, using the last acceptable position to fill missing data points. Location analysis is performed over the filtered GPS dataset to identify the participant's home, time spent at that location and occurrences of trips outside their home. Further processing is completed to identify outside-of-home locations based on GPS data clusters. The number of destinations and distance from home of these clusters are computed and transit between those locations are classified into two categories based on filtered velocity for each transit: being in a vehicle or on foot. Finally, a minimal area ellipse is created to include all the GPS positions of the participant's dataset. From these processing steps, 12 outcomes are extracted and can be separated into two categories: location and activity outcomes (Table 3) or transit and community mobility outcomes (Table 4). Location and activity outcomes include: Time at home ratio (THR), Trips out (TO), Destinations (D), Maximal distance of destinations (MDD), Active time ratio (ATR), and Steps (S). Transit and community mobility include: distance in a vehicle (DV), time in a vehicle (TV), distance on foot (DF), time on foot (TF), ellipse area (EA), and ellipse maximum distance (EMD).

**Table 1:** Signal processing steps applied to accelerometer data collected.

**ACCELEROMETER DATA**	
**Preprocessing**	The timestamps from accelerometer data are extracted and used to split the raw data file into days to build an initial time vector.
**Active time detection**	The observed subject is considered to be active if the filtered (f) 3D acceleration vector values exceed a specified threshold (min_g) for a minimum time ratio (ratio) computed with a rolling window (w). Filter parameters (f) = low-pass filter at 5 Hz and high-pass filter at 1 Hz, minimal acceleration vector value (min_g) = 0.015 g, minimum time ratio to consider the subject active = 50% of the window length, rolling window parameters (w) = length: 10 s, 50% overlap.
**Step detection**	A step detection algorithm is applied to active time epochs identified and uses a hybrid design consisting of frequency analysis and impact detection. Consider that normal walking speed of healthy individuals generates acceleration peaks of approx. 2 times/s (2 Hz), an aggressive filter (fcut 3 Hz) is applied to the norm of the triaxial accelerometer signal. Using a Fast Fourier Transform (FFT) any remaining power in the 0–3 Hz bandwidth in the data epoch is consider as containing potential steps. Selected data epoch is sent to an Impact Detector (ID) to count accelerometer spikes. The ID uses interpeak minimum distance (150 ms) and minimum height (0.05) to identify peaks and the threshold detection is applied on the envelope of the signal rather than raw norm vector to remove noise artifacts associated with individual impacts (i.e., several peaks generated for a single impact).

**Table 2:** Signal processing steps applied to GPS data collected.

**GPS DATA**	
**Preprocessing**	The timestamps from GPS data are extracted and used to split the raw data file into days to build an initial time vector.
**Filtering and data interpolation**	Each GPS position with a reported precision less than the specified GPS precision (gp) value is filtered and removed from the dataset. The minimum gp = 5 m. For each point in the continuous timeseries without a GPS position (because of filtering or no position), a zero-order hold interpolation is used to complete the series so that each missing value is reported as the last valid position.
**Clusters and transit identification**	A temporal cluster is created when time-consecutive positions are within close proximity to each other, over a specific window frame. Adjacent clusters are then merged to form a larger cluster. A rolling window (length, overlap) is used to identify temporal clusters. A window is considered to form a cluster if the (1-γ) ^th^ quantile distance from the median spatial center of the window is less than a specified radius (r). Window length = 300 s. Window overlap = 50% γ value = 0.3 Maximum distance radius (r) = 30 m.
**Transit classification**	For each transit between two clusters, the type of activity is identified. Positions comprised in a transit period are considered to be in a vehicle if the RMS speed over a 90s period ≥ 10 km/h. Positions in a transit period not considered to be in a vehicle are classified as on foot.
**GPS time series ellipse modeling**	The minimum span ellipse that can fit all of the positions of the dataset is computed using a minimum covariance estimator. The ellipse does not have to be centered on home, as it encompasses the whole dataset.

**Table 3:** Definition of location and activity outcomes.

**LOCATION AND ACTIVITY OUTCOMES**	
**Active time ratio**	Sum of data points classified as active over the total recording period expressed in minutes.
**Steps per recording**	The number of steps detected divided by the number of recording days expressed in number of steps per day.
**Time at home ratio**	Sum of data points identified within the home cluster over the total recording period expressed in minutes.
**Trips out**	The number of trips outside home per day is classified when the observed subject leaves the home cluster and returns at a later time in the same day.
**Destination trips**	The number of clusters reached in each trip over the total recording period per day.
**Max dist. destination**	Max distance traveled to reach destination cluster outside of the home cluster over the total recording period expressed in kilometers.

**Table 4:** Definition of transit and community mobility outcomes.

**COMMUNITY AND MOBILITY OUTCOMES**	
**Distance in vehicle**	Distance computed from all the data points over the total recording period which are not part of a cluster and identified as being in a vehicle.
**Time in vehicle**	Time computed from all the data points over the total recording period which are not part of a cluster and identified as being in a vehicle.
**Time on foot**	Time computed from all the data points over the total recording period which are not part of a cluster and identified as being on foot.
**Distance on foot**	Distance computed from all the data points over the total recording period which are not part of a cluster and identified as being on foot.
**Ellipse max distance**	Length of the major axis of the minimum span ellipse of the dataset over the recording period. This variable is transformed into log value.
**Ellipse area**	Geometric 2D area of the minimum span ellipse of the dataset over the recording period. This variable is transformed into log value.

**Figure 2:**
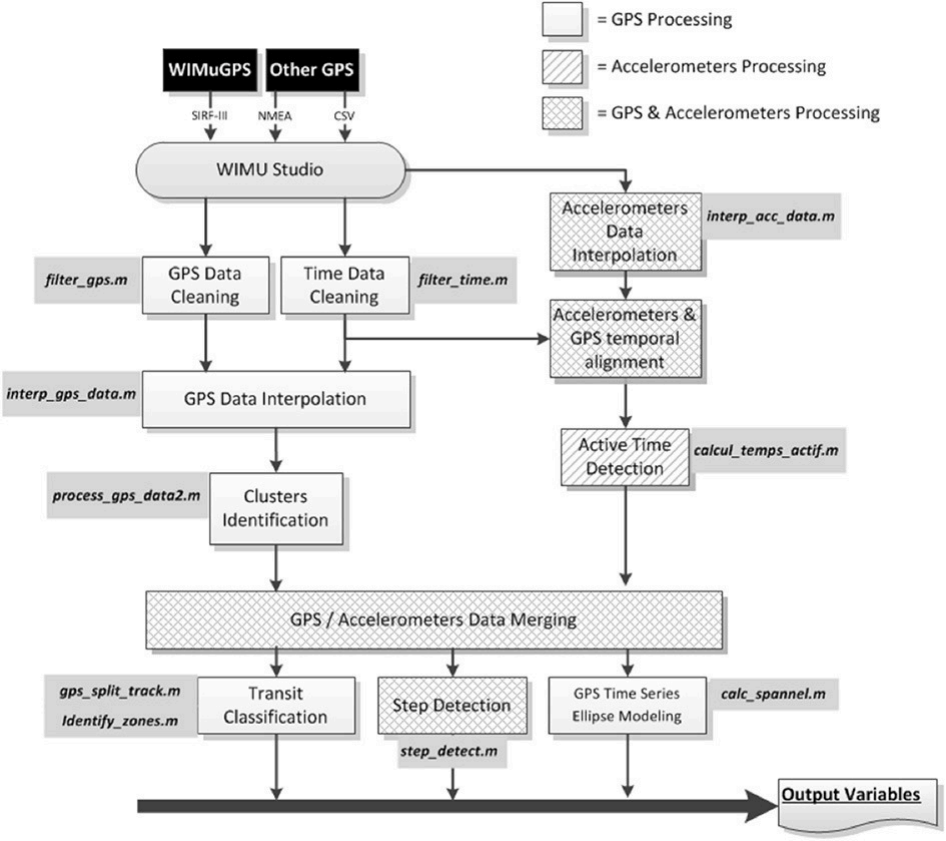
Analysis workflow.

Figure 2 presents the analysis workflow. An in-house developed open-source software, WIMU Studio (https://github.com/introlab/openwimu) was used as a first step in the data analysis process to format data coming from the different sensors (WIMUGPS, QStarz) in a standard file format and to split each day in its own file. Afterwards, data processing required to compute the output variables was done in Matlab R2015b using the mapping toolbox for geospatial computations. Each data processing step refers to a.m Matlab script file. Details of the signal processing and the algorithms used to compute each output variable can be found in specific.m Matlab script source code made available as Supplemental Files to this manuscript.

### Data analysis and statistics

Accuracy, compliance, and their impact on data loss are inherently known issues when collecting data with wearable systems under free living conditions. To ensure that each participant's data from which the activity and mobility outcomes were extracted and synthesized adequately represented daily behaviors of that participant and that a sufficient number of days were captured to establish a meaningful profile of behaviors across multiple days, quality control criteria were applied to the collected dataset (*n* = 75). Each day of recordings was required to have a minimum of 480 min of recorded data and a compliance of 55% (6 out of the 11 days) to be kept for processing. Additionally, each day of data was reviewed individually to determine if the devices were worn. Repeated (*n* = 2) days with no GPS signal recordings were considered an anomaly and were removed from the individual dataset. Days with < 30 min of active time were noted, and upon examination of the accelerometer signals activity and expected orientation of the device, periods of non-wear during the day were identified. Additionally, days in which non-wear periods totaled > 120 min were excluded from the individual dataset. Descriptive statistics (mean, SD, range) and normality of the data for each variable were computed. Anormal distribution (*p* < 0.05, Shapiro-Wilk) were inspected visually to confirm the result. Depending on the normality of the distribution, independent *t*-tests or non-parametric Mann-Whitney U tests were performed to assess group differences in activity and mobility outcomes according to the proposed classifiers for individuals' functional impairment and environmental variables. Non-parametrics correlations (Spearman rho) between activity and mobility outcomes and lifespace assessment total scores were also computed. The significant level was set at *p* < 0.05. All analyses were performed on SPSS 23.

## Results

### Personal, functional and environmental outcomes

**Figure 3:**
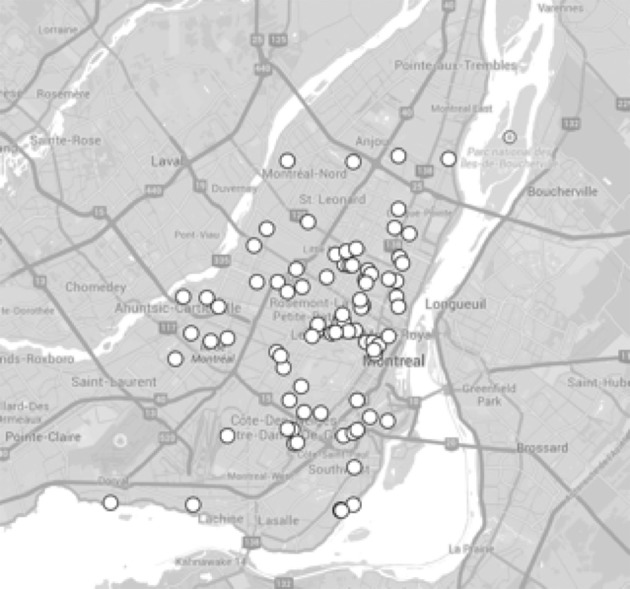
Location and dispersion of the participants on the island of Montreal.

After applying the above quality control criteria, the original data set was reduced from 75 to 54 participants (28% attrition). The location and dispersion of the participants on the island of Montreal (agglomeration of 1,886,481 people) are illustrated in Figure 3. A majority (80%) of participants reported living >4 years in a condominium or apartment with an average of 2.2 bedrooms.

The mean and standard deviation (SD) of the daily recording duration for the final sample was 723 min (about 12 h) ± 99 min. Activity and mobility outcomes were extracted and a mean number of valid recording days of 9.52 ± 3.23 found. The characteristics of the final sample of participants (*n* = 54) are presented in Table 5. The sample consisted of 26 men and 29 women aged between 55 and 85 (mean of 66 years ±7 years). Most were highly educated, with less than 20.4% not finishing high school. Additionally, the sample had a mean annual reported income of 37$k (23% of the sample with an annual income categorized as low income by Statistics Canada) and an average BMI over the 25 kg/m^2^ threshold (typically used as an indicator of an overweight body mass composition). More than 75% of the sample had no sign of impairments related to pain, cognition or depression. With regards to living situation, 60% of participants lived with someone while in the workplace, 20% were still active professionally. Furthermore, over a third (35%) of the participants had no vehicle access, however, the average walking time to shops and services for the 12 resources cited was approximately 11 min, which implies a relatively dense urban area with easy access to services and shops.

**Table 5:** Descriptive statistics for personal, functional and environmental variables for sample of participants (*n* = 54).

**Variables**	**Mean**	**SD**	**Classifier**	**Percent (%)**
Age (M)	65.7	6.6	Age (>75 years)	11.1
Age (F)	65.2	6.2	Gender (M)	54
Education (years)	15.7	3.1	Educ. (<highschool)	20.4
Income ($ 1000's)	37.0	23.7	Income (<$20,000)	22.6
BMI (kg/m^2^)	24.2	3.3	BMI (overweight >25)	27.9
Pain Past 30 Days (score /4)	1.4	0.7	Pain (impacting ADL in past 30 days)	22.2
Cognition score on MOCA (/30)	27.1	2.4	MOCA (impaired cognition)	24.1
Depression score on GDS (/10)	3.0	2.7	GDS (depressive symptoms)	24.5
Living Situation (alone, with someone)	N/A	N/A	Liv. Sit. (live alone)	40.7
Professional Status (retired, working)	N/A	N/A	Prof. Stat. (retired)	79.6
Vehicle Access (access to a car or not)	N/A	N/A	Veh. Acc. (no access to a vehicle)	35.2
Average walking time to services (mins)	11.2	4.8	Walk Acc. (>15 min walk from services)	24.1

### Community activity and mobility outcomes

**Figure 4:**
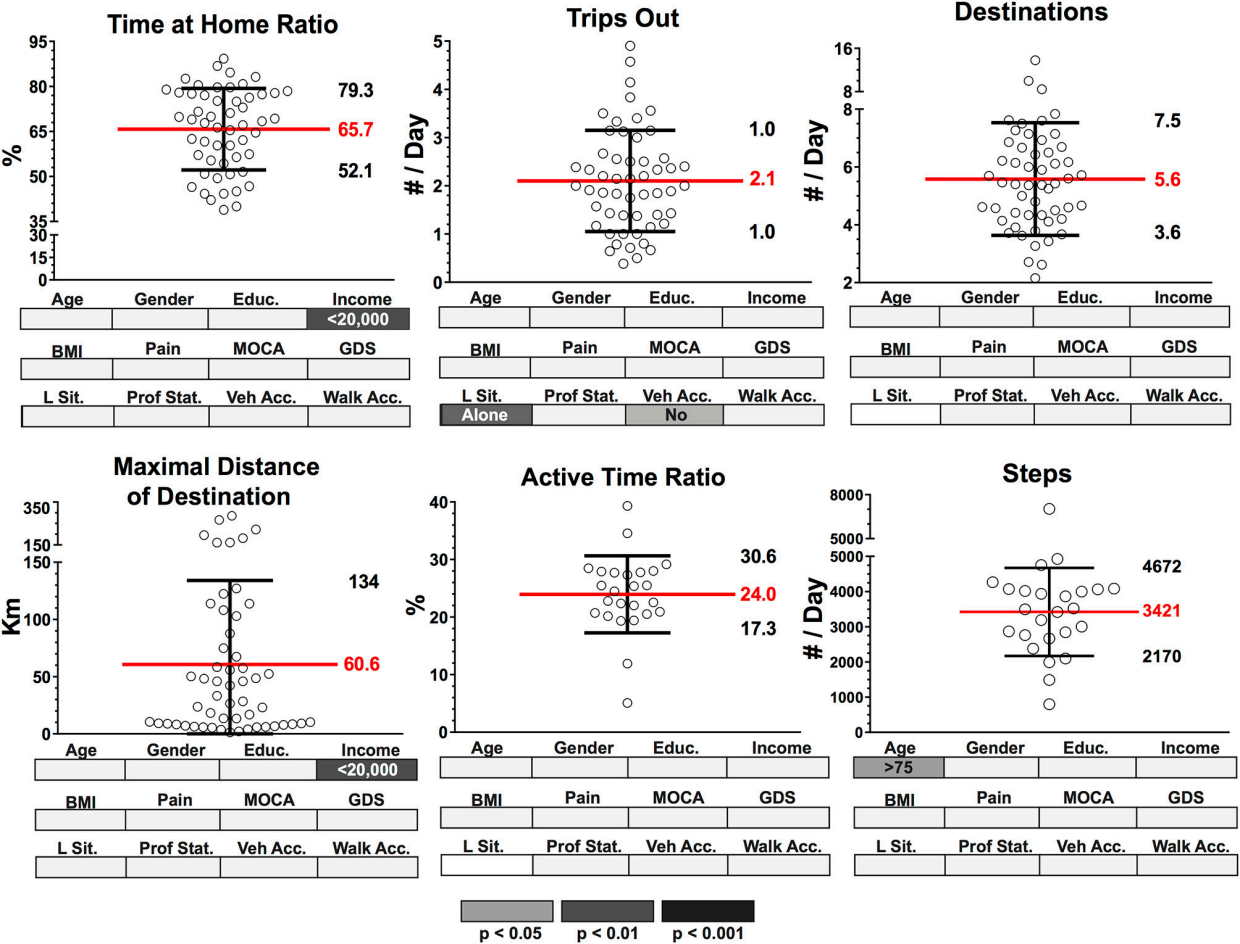
Dispersion (mean and standard deviation) of group data (*n* = 54) for location and activity outcomes based on GPS and accelerometer data. Group data for each outcome were segmented according to known classifiers: Age (>/<75 years), Gender (M/F), Education (>/<high school level), Income (>/<20,000 per year) body mass index (BMI) (>25 = overweight, <25 = healthy), Pain (pain in the past 30 days affecting ADL, pain free), MOCA (MOCA score indicating cognitive impairment, normal cognition), GDS (GDS score indicating presence of depressive symptoms, normal); Living Sit. (Living Situation: alone, with someone), Prof. Stat. (Professional Status: working, retired), Veh. Acc. (Vehicle Access: have vehicle access, do not have vehicle access), Walk Acc. (Walking Access: within 15 min of shops and services, >15 mins) Significant group differences (*p* < 0.05, *p* < 0.01, *p* < 0.001) for each classifier after independent *t*-tests are highlighted below each dispersion graph in gray scale density code.

Dispersion (mean and standard deviation) of group data (*n* = 54) for location and activity outcomes based on GPS and accelerometer data are presented in Figure 4. An average time at home ratio (THR) throughout the recording period was 65.7 ± 13.6%, with those classified as having a low income having a significantly greater percent of time spent at home compared to those whose income was above the poverty line for the Island of Montreal. Participants made an average of 2.1 ± 1.1 trips out (TO) per day with 5.6 ± 1.9 destinations (D) reached during these trips. Individuals who lived alone or did not have vehicle access completed significantly less number of trips. The maximal distance of destinations (MDD) varied among participants, with an average of 60.6 ± 73.4 km among the sample. Within the sample, individuals who had a low income traveled a shorter distance to their destinations compared to those with greater incomes. Participants' active time ratio (ATR) per day was less than their THR ratio, with an average of 24 ± 6.7% of each recording day consisting of active time. The average number of steps (S) taken by participants each day 3421 ± 1251 with older participants (>75 years of age) taking fewer steps on an average day.

**Figure 5:**
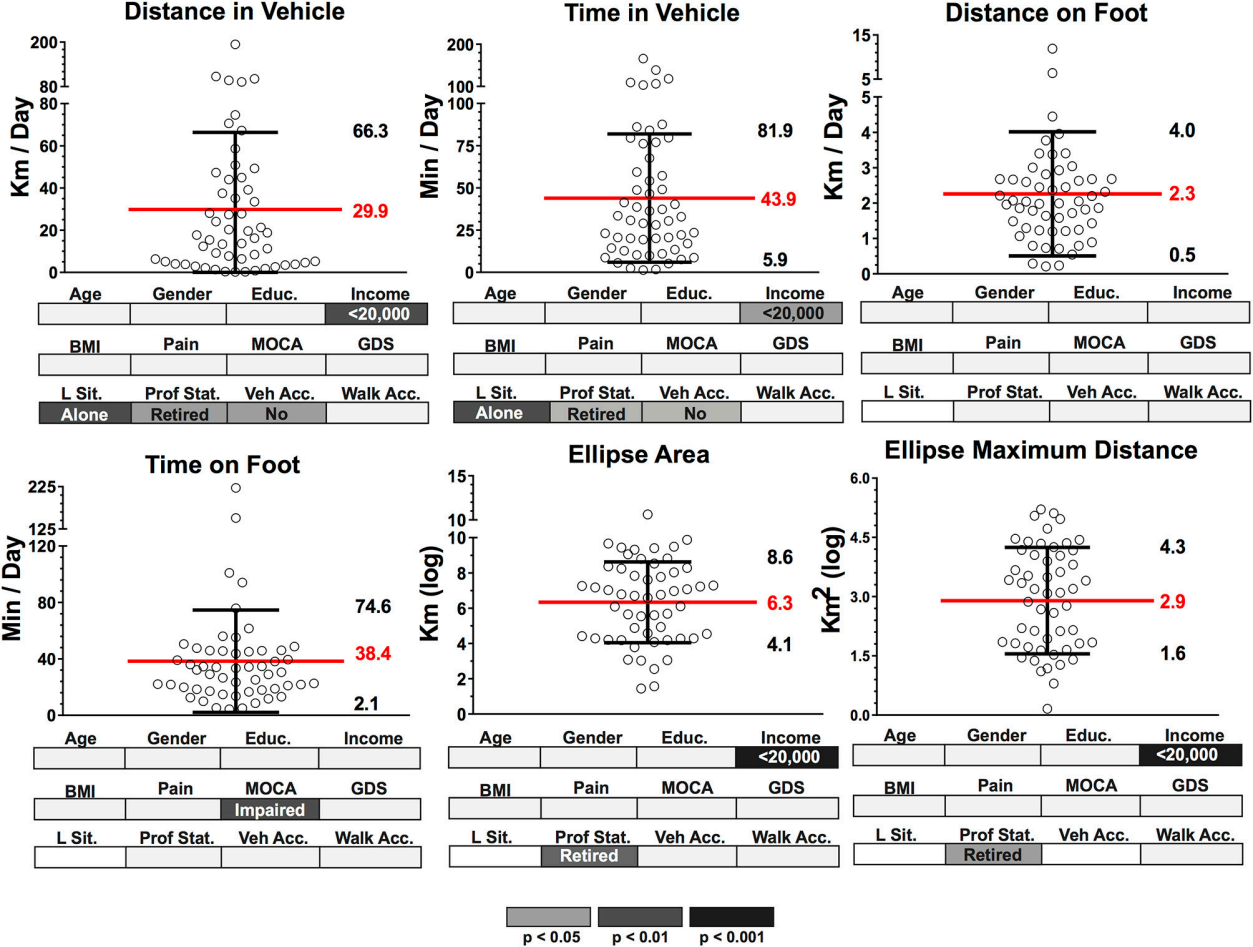
Dispersion (mean and standard deviation) of group data (*n* = 54) for transit and community mobility outcomes based on GPS and accelerometer data. Group data for each outcome were segmented according to known classifiers: Age (>/<75 years), Gender (M/F), Education (>/<high school level), Income (>/<20,000 per year), body mass index (BMI) (>25 = overweight, <25 = healthy), Pain (pain in the past 30 days affecting ADL, pain free), MOCA (MOCA score indicating cognitive impairment, normal cognition), GDS (GDS score indicating presence of depressive symptoms, normal); Living Sit. (Living Situation: alone, with someone), Prof. Stat. (Professional Status: working, retired), Veh. Acc. (Vehicle Access: have vehicle access, do not have vehicle access), Walk Acc. (Walking Access: within 15 mins of shops and services, >15 min) Significant group differences (*p* < 0.05, *p* < 0.01, *p* < 0.001)) for each classifier after independent *t*-tests are highlighted below each dispersion graph in gray scale density code.

Dispersion (mean and standard deviation) of group data (*n* = 54) for transit and community mobility outcomes based on GPS and accelerometer data are presented in Figure 5. On average, participants' distance in vehicle (DV) was 29.9 ± 36.4 km and 43.9 ± 38 min on average per day. The distance traveled by and time spent in a vehicle was significantly lower in the lower-income population and also significantly different for those who lived alone, were retired or without vehicle access. Additionally, distance on foot (DF) and time on foot (TF) were 2.3 ± 1.8 km and 38.4 ± 36.2 min per day respectively. TF for those with impaired cognition (according to MoCA score) was significantly shorter per day. Lastly, ellipse area (EA) and ellipse maximum distance (EMD) were 6.3 ± 2.3 km (log) and 2.9 ± 1.4 km (log). Both EA and EMD were significantly different in those who had a low income and in those who were retired.

### Relationship between activity and mobility outcomes and LSA scores

**Figure 6:**
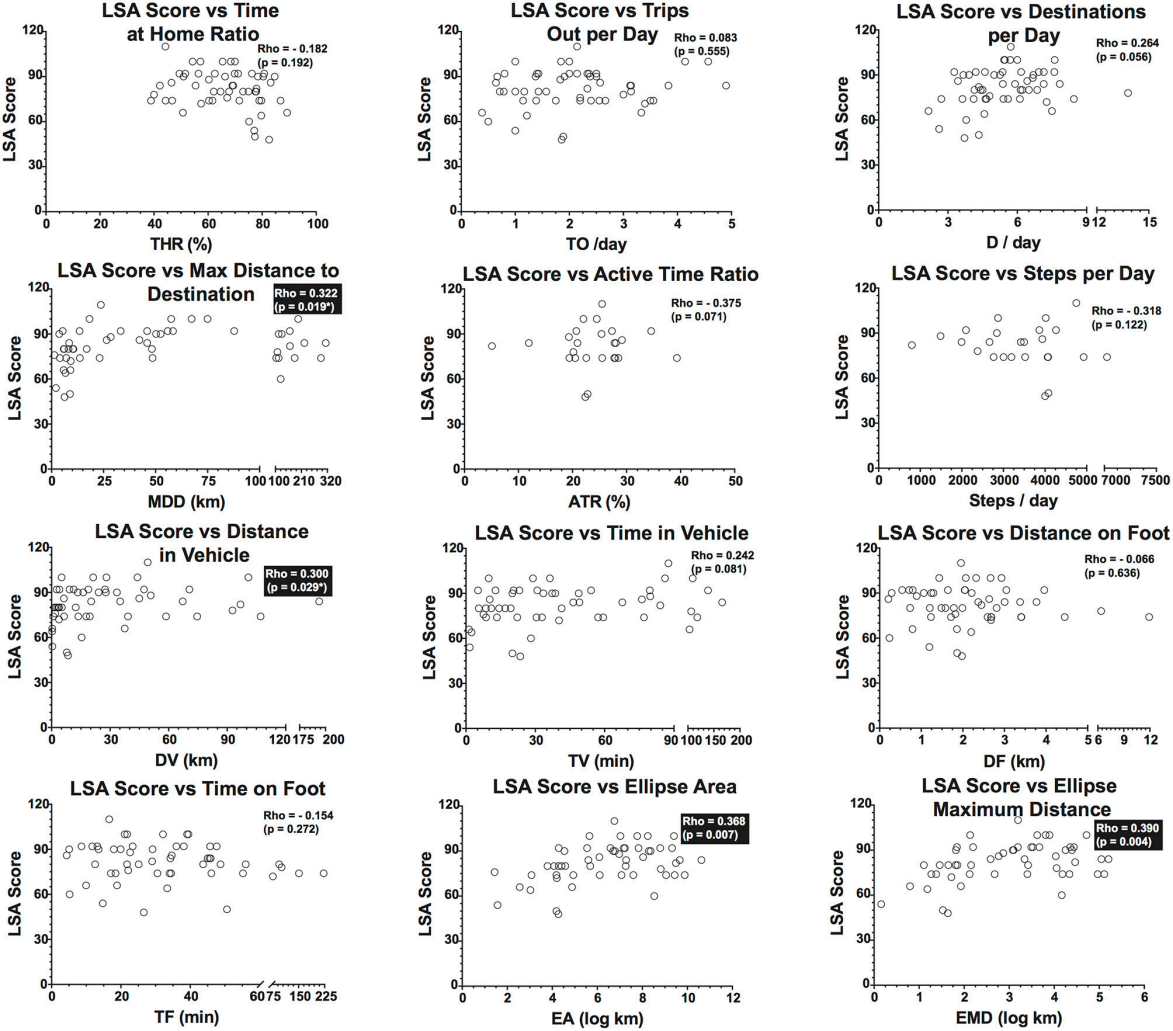
Dispersion of data points, Spearman Rho correlation coefficients and *p*-values between LSA (Life Space Assessment) total score and Location and Activity and Transit and Community mobility outcomes. Significant correlations are highlighted by boxes.

Significant but weak correlations were found between the participants LSA scores and only four activities and mobility outcomes seen in Figure 6. Of the location and activity outcomes, maximum distance of destination was the only variable with a significant relationship (*p* = 0.019). Transit and community mobility outcomes had significant relationships for distance in vehicle (DV; *p* = 0.029), ellipse area (EA; *p* = 0.007) and ellipse maximum distance (EMD; *p* = 0.004) with LSA scores /120.

## Discussion

Free living activity and community mobility were assessed using wearable sensors in healthy older adults without any mobility impairments. It should be noted that we applied strict quality control to the data collected which reduced the initial sample from 75 to 54 participants (28% attrition). Our data loss is thus reported as participants' dataset not meeting our quality criteria. The rationale behind this decision was to ensure that the computed outcomes were derived from a representative time portion of a typical day (minimum of 8 h of recordings during each day) and that we captured a sufficient number of days to provide a good representation of activity and mobility behaviors over a long period (close to 1 week which includes potentially a weekend). Data loss reported in the literature for similar measurement approaches are less, however most of those studies don't use such strict quality criteria as those applied here. It is noticeable that the data loss in this study affected our power to analyze the proposed outcomes and warrant an explanation of the difficulties associated with the use of wearable sensors in free-living conditions in older adults.

Wearability, defined as “the physical ability to mount a device on the body or the physical and mental comfort of the wearer” (61) and usability, defined as “the extent to which a product can be used by specified users to achieve specified goals with effectiveness, efficiency, and satisfaction in a specified context of use' (62) are key factors to minimize data loss when using platforms to capture behavior and activity data over long periods. Wearability is often a function of form, which is primarily determined by the battery of the wearable system and its autonomy to power the sensors according to the scenario envisioned (sampling frequency of sensors and duration of recordings without charging it). While there are multiple ultra-low power sensors (mostly motion sensors such as accelerometers) available, sensors that require communication via radio-frequency like a GPS receiver consume over 6,000 times more energy. As a result, when using a GPS receiver continuously at a rate of 1 Hz, it is near impossible to reduce the size of the battery (and the device) in order to increase its wearability without compromising the recording duration. Consequently, this also affects the location of where the device can be worn. In our study, we recorded continuously and used systems with clips that were worn on the hip (with or without a belt). Wearability was thus not optimal due to the fact that the devices needed to be recharged every night. This affected wear compliance and reliability of the recordings (i.e., device worn but dead battery or battery dying during the day. The solution to these wearability and usability limitations requires compromising the use of continuous sampling of GPS data (thus affecting the measures of transportation modes) or will require implementation of intelligent power management of the GPS sensor. This method would ensure that the device is only powered when certain conditions are met (e.g., the person is outside) and when its duty cycle is pushed to the minimum (i.e., power up and down cycles are optimized to keep data acquisition computing cycles to compute satisfactory GPS data). However, although this can be done with research devices, it is highly unlikely to be applied to commercial devices that are designed for specific consumer use (e.g., tracking runs of 3–5 h).

Results on the remaining sample (*n* = 54) showed that on average in a typical day, participants spent about 34% of the recording time outside of the home, with about two trips outside of the home and approximately six destinations reached in total. Typical transit distance per day was 30 km on average with 44 min spent in a vehicle. Approximately 2.3 km of travel was on foot, which was a time of 38 min on average per day. The values for the ellipse area and ellipse maximal distance of mobility over the recording period were highly variable. The values for the time spent at home may be overestimated with respect to other observations reported in previous literature (37–39), as our cluster-based processing approach (buffer zone around the cluster) and method of computing time at home (time spent in the cluster where there was the most data points occurred in a day) could have been affected by certain participants who spent most of their day outside of their home in a cluster that was not in fact their home (i.e., those still working or visiting outside of their home). This could be addressed by fixing a known coordinate as the home location for later use during data analysis. Furthermore, while the sensing devices and signal processing approaches are different, the travel behavior results (trip out and destination) presented are similar to those reported by others (40–42), suggesting that these outcomes are relatively robust and reproducible for a sample of healthy older adults. The mean maximal distance achieved over the recording period was highly variable (mean of 60 km with SD of 134 km) which potentially highlights certain motivations and opportunities to travel such distance. For example, one outlier used a personal car to travel to their workplace close to 150 km from his home.

Another limitation of the extraction method was that the active time ratio and steps were computed only for the participants who wore the WIMU-GPS. The mean active time ratio was transformed in minutes per time of recording which corresponds to about 3 h of activity per day. The approach used to compute active time in our participants from accelerometer data is based on a threshold and temporal density of the acceleration signals that was developed based on observations of older adults during in-patient rehabilitation sessions (63). Therefore, active time is a relatively sensitive measure that captures sustained low level physical activity with a small-time window which does not classify the context or the intensity of the activity. The number of steps per day (average of 3,421 steps per day, with a range between 950 steps and 7,500 steps) computed during these active time periods was relatively low in comparison to studies reported in the literature on the number of steps taken by healthy older adults. Counting steps using accelerometer data is not trivial as the devices used, their location (wrist, hip, trunk), the underlying algorithms and their specificity to detect certain activities in certain populations, affect the accuracy and validity of the steps counted, thus making comparisons difficult. This is clearly still an unresolved issue, as shown by the recent systematic review published on activity trackers (32). With the low number of steps taken on an average day, it can be deduced that our sample of healthy older adults was mostly sedentary (fewer than 5,000 steps per day). While the accuracy of our measurement approach and algorithm (an ongoing validation study) cannot be attested to, the combination of computing steps for time epochs that were considered active, the location of the sensor (at the hip) and the proposed hybrid algorithm were choices made to maximize counting real steps and minimize artifacts.

The construct validity of our outcomes was explored using personal, functional, and environmental variables to see if the activity and mobility outcomes derived from accelerometer and GPS data recordings differed among variables. The idea of life space constriction with aging is mostly based on the rationale that increasing vulnerability to negative environmental aspects is linked to deteriorating functional ability among the elderly as well as the idea that disease states (heart disease, neurological disease, arthritis/neuralgia) contribute to this phenomenon. While there were important inter-individual variations in the lifespace measured using the area of the ellipse and the maximal distance of the ellipse outcomes computed from the GPS data—outside of a few personal variables such as income and professional status—no other variables were associated with a decreased lifespace. This can be explained in part by the population selected in our study (healthy older adults without any mobility impairments) and the density of the urban area they lived in. Although certain personal variables (income, living situation, vehicle access, professional status) appeared to affect some of the measured outcomes, functional status and environmental variables did not. Age and walking access to shop and services did not impact the majority of the measured outcomes (with the exception of the number of steps). Although this was surprising, it could possibly be explained if one considers the population recruited as well as the fact that this group lived in a dense urban area. Thus, the effect of these variables appears to be negligible. Vehicle access impacted the transit distance and time in a vehicle but not the overall lifespace, which suggests that in an urban area such as the Island of Montreal, other means of active transportation can mitigate or facilitate the overall lifespace of an individual. Furthermore, amongst personal variables, participants categorized as having a low income spent more time at home and had lower maximal distances of destination. They also had a more constrained lifespace as measured by the ellipse area and maximal ellipse distance outcomes. This could potentially be explained by socio-economic status, which has been demonstrated to impact lifespace and can be directly related to access to transportation and social participation and opportunities.

With regards to the Lifespace assessment scores and the 12 location, activity and community mobility outcomes, only four of twelve correlations were significant (spearman rho < 0.40, *p* < 0.05). Therefore, although the maximum distance of destination, distance in vehicle, ellipse area and ellipse maximum distance were related to the LSA scores and appear measure the same construct, these correlations are weak, if at all significant. Consequently, it could be hypothesized that measures of lifespace provide a different overall representation of daily activity and lifespace compared to the presented method using wearable technology. An explanation for the lack of a likeness between the two measures could be the fact that the LSA provides data for the preceding 4 weeks and the GPS and accelerometer only 2 weeks (worn starting at day 14). Then, this could have resulted in discordance between actual performance (location, activity and community mobility outcomes) and self-reported lifespace, which has also been reported in various studies (64–66). Indeed, while the LSA is the most commonly used instrument to measure lifespace, it also has considerable limitations, the most important being that the LSA relies heavily on recall and honest reporting (27). This recall is for a lengthy 4-week period and thus is especially problematic for many, as trying to identify occurrences and frequencies of specific events in the different lifespace levels is difficult. Therefore, this measure is only an approximation of averages. To what extent these averages sufficiently represent actual mobility of the individual remains to be established. Another drawback of the LSA is that its score fails to account for the duration of mobility bouts or time spent in each lifespace level. In addition, the lifespace levels used with the instrument are categorized on a 5-level scale representing a coarse representation of existing real lifespaces. A concern is that these lifespaces are delimited by virtual boundaries which are subject to individual judgement (e.g., interpretation of the boundaries of one's neighborhood may differ between individuals). Furthermore, lifespace measures may not apply to rural areas due to substantially differing mentalities concerning what neighborhoods and other lifespace levels encompass. Therefore, these limitations may contribute to the discordance and lack of correlation with the extracted outcomes using the proposed method.

## Conclusion

The prevalence of mobility limitations with aging is high and their impact on activity and community mobility require new so-called “ecological measures—i.e., measures that consider the environment of the person” to understand mitigating personal, functional and environmental factors that affect physical, psychological and social aspect of an older adult's life. Aactivity and community mobility measurements based on GPS and accelerometer sensor data can offer unique insights on this dynamic. Activity and mobility profiles from the proposed approach are highly variable and distinct in healthy young older adults and some of these variations are linked to personal characteristics. The construct validity of the proposed approach appears promising but requires further studies directed at populations with mobility impairments. Wearability and usability of the devices used to record the data affect compliance and data quality.

## Author contributions

PB developed the analytical methods and metrics, conceived the experiment, analyzed the data and drafted the manuscript. MB participate in the data collection, interpretation and reviewed the paper. SB developed the analytical methods, analyzed the data and reviewed the paper. CD conceived the experiment and supervise the data collection, helped in the the interpretation of the data, and reviewed the paper.

### Conflict of interest statement

The authors declare that the research was conducted in the absence of any commercial or financial relationships that could be construed as a potential conflict of interest.
